# In situ atomic-scale imaging of the metal/oxide interfacial transformation

**DOI:** 10.1038/s41467-017-00371-4

**Published:** 2017-08-21

**Authors:** Lianfeng Zou, Jonathan Li, Dmitri Zakharov, Eric A. Stach, Guangwen Zhou

**Affiliations:** 1 Department of Mechanical Engineering & Materials Science and Engineering Program, State University of New York at Binghamton, Binghamton, NY 13902 USA; 2 Department of Physics, Applied Physics and Astronomy & Materials Science and Engineering Program, State University of New York, Binghamton, NY 13902 USA; 3 Center for Functional Nanomaterials, Brookhaven National Laboratory, Upton, NY 11973 USA

## Abstract

Directly probing structure dynamics at metal/oxide interfaces has been a major challenge due to their buried nature. Using environmental transmission electron microscopy, here we report observations of the in-place formation of Cu_2_O/Cu interfaces via the oxidation of Cu, and subsequently probe the atomic mechanisms by which interfacial transformation and grain rotation occur at the interfaces during reduction in an H_2_ gas environment. The Cu_2_O→Cu transformation is observed to occur initially along the Cu_2_O/Cu interface in a layer-by-layer manner. The accumulation of oxygen vacancies at the Cu_2_O/Cu interface drives the collapse of the Cu_2_O lattice near the interface region, which results in a tilted Cu_2_O/Cu interface with concomitant Cu_2_O island rotation. These results provide unprecedented microscopic detail regarding the redox reactions of supported oxides, which differs fundamentally from the reduction of bulk or isolated oxides that requires the formation of new interfaces between the parent oxide and the reduced phase.

## Abstract (web summary)

Metal/oxide interfaces play an important role in heterogeneous catalysis and redox reactions, but their buried nature makes them difficult to study. Here, the authors use environmental transmission electron microscopy to probe the atomic-level transformations at Cu_2_O/Cu interfaces as they undergo redox reactions.

## Introduction

Many reactions such as oxidation and reduction, heteroepitaxial growth, and heterogeneous catalysis involve the formation of metal/oxide interfaces, and the resulting interfaces are a critical region in controlling both reaction mechanisms and macroscopic properties. For instance, metal/oxide interfaces act as active sites that enhance catalytic reactivity due to synergy between the support and supported phases^1–4^. Metal/oxide interfaces also serve as a reservoir for oxygen during oxidation, and thus significantly alter the oxidation kinetics^5^. Finally, the nature of the heteroepitaxial alignment between metal nuclei and the metal oxide substrate controls the morphology of the epilayer during thin-film growth^6–8^. Understanding the processes that occur at the metal/oxide interface is crucial for a full mechanistic understanding of many processes and phenomena, as this is where new metal/oxide interactions originate, and thus for further advancing applications that rely on the atomic-level control of the metal/oxide interface.

Unfortunately, probing interfacial dynamics in situ has always been a major challenge, mainly because of the experimental inaccessibility of buried interfaces. Generally, transmission electron microscopy (TEM) offers the opportunity to study static interfaces, but this usually requires complex sample preparation and suffers from the possibility of introducing artifacts and contamination to the interface region. In relation to technologically relevant process such as oxidation/reduction, catalysis, and thin-film growth, metal/oxide interfaces are in fact highly dynamic in their response to and interaction with the environment. Fundamental understanding of interface dynamics not only requires resolving the local structure at the atomic scale, but also the ability to capture this structural evolution in real time and under reaction conditions. TEM has evolved dramatically in recent years and allows for temperature-, time-, and pressure-resolved imaging of gas-surface reactions by introducing a reactive gas to the sample while simultaneously monitoring the structural evolution at the atomic scale^9–16^.

Cu-based materials are widely used in different oxidizing/reducing environments for a wide variety of applications, including Cu interconnect technology and heterogeneous catalysis. With all these applications, the exposure of Cu to oxygen results in the generation of a Cu oxide and thus a metal/oxide interface. Switching to hydrogen gas leads to the reduction of the metal oxide. These redox conditions are typical of a number of catalytic gas--surface reactions, such as the water-gas-shift reaction^17^, CO oxidation^18^, and methanol oxidation^19^ as well as catalyst regeneration treatment^20^ for which H_2_ (or CO) is involved either as a reactant or a reaction product.

In this work, we employ environmental TEM to observe the structural evolution of Cu_2_O/Cu interfaces at the atomic scale and in real time in a reducing environment inside the microscope. We have chosen to study the Cu_2_O/Cu interface as a model system, in order to probe both interface stability and the phase transformations that occur in the reducing environment. We show that the H_2_-induced Cu_2_O reduction results in the transformation of Cu_2_O → Cu via step-flow at the epitaxial Cu_2_O/Cu interface. While the epitaxial metal/oxide interface is maintained initially, we find that the transition from an epitaxial to nonepitaxial interface occurs via grain rotation driven by the accumulation of oxygen vacancies at the interface region.

## Results

### In situ creation of Cu_2_O/Cu interfaces

**Fig. 1:**
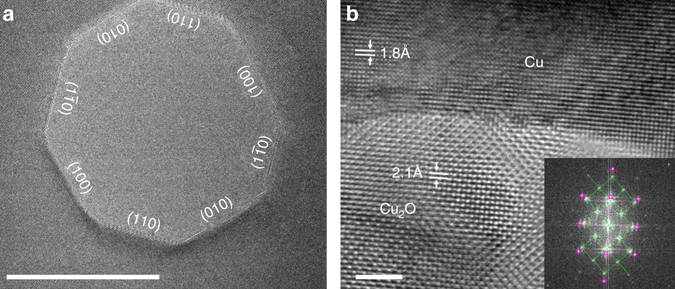
In situ formation of Cu_2_O/Cu interfaces. **a** TEM image of an as-prepared nano-hole in the Cu(100) thin film after annealing at 600 °C in the flow of H_2_ gas at *p*H_2_ = 10^−3^ Torr. **b** HRTEM image of a Cu_2_O/Cu interface formed by oxidizing a faceted Cu edge to form a Cu_2_O layer at 350 °C and *p*O_2_ = 5 × 10^−3^ Torr for 5 min. The *inset* to the bottom-right corner is a diffractogram of **b**, where the *green lines* and *purple rings* illustrate the diffraction spots for Cu_2_O and Cu phases, respectively. *Scale bar*, 20 nm **a**, 2 nm **b**

The first step of the study is to create Cu_2_O/Cu interfaces, which is achieved through oxidizing a single-crystal Cu(100) thin film (nominal thickness of ~50 nm, see Methods section) inside the TEM. The Cu thin film is first heated at 600 °C in flowing H_2_ gas (*p*H_2_ = 10^−3^ Torr) to generate holes with well-defined and atomically clean {100} and {110} facets, as shown in Fig. 1a. The annealed sample is then oxidized at 350 °C to form Cu_2_O/Cu interfaces by introducing oxygen gas at *p*O_2_ = 5×10^−3^ Torr. Figure 1b shows a typical HRTEM image of a Cu_2_O/Cu interface generated by the oxidation of a Cu-faceted edge. In situ TEM observations of the Cu_2_O/Cu interface dynamics are made in the cross-sectional view.

Oxidation of Cu can produce various phases of Cu oxides, including Cu_2_O, Cu_4_O_3_, and CuO^21^. Thus, identification of the oxide phase that has formed is necessary before investigating the subsequent interfacial reactions that occur during reduction. Measurements of the periodicity and spacing of atomic columns in both the Cu substrate and oxide regions, along with the diffractogram of the whole area, show that the Cu_2_O phase is formed, with the epitaxial relation of Cu_2_O(200)//Cu(200) and Cu_2_O[001]//Cu[001] (Supplementary Note 1). The HRTEM analyses above confirm that an epitaxial Cu_2_O layer is formed along the Cu (100) facet.

### Cu_2_O → Cu interfacial transformation

**Fig. 2:**
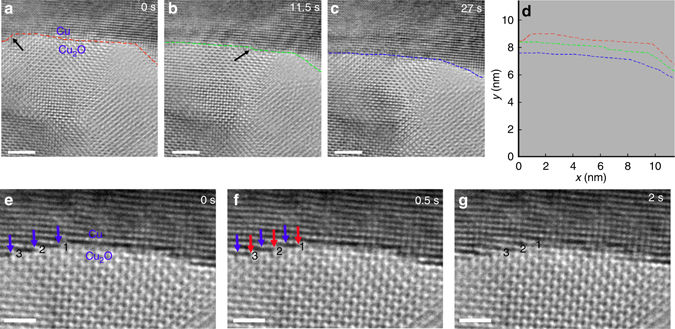
Cu_2_O→Cu interfacial transformation. **a**–**c** HRTEM snapshots (Supplementary Movie 1) showing the Cu_2_O→Cu conversion at the Cu_2_O/Cu interface at *T* = 350 °C and *p*H_2_ = 4 × 10^−2^ Torr, the *colored dash lines* outline the Cu_2_O/Cu interface, the *black arrows* mark the interface steps. **d** Traces of the position of the Cu_2_O/Cu interface at the different times show that the interface moves toward the Cu_2_O layer. **e**–**g** High magnification view of the dynamics of the Cu_2_O→Cu interfacial conversion at *T* = 350 °C and *p*H_2_ = 4 × 10^−2^ Torr (Supplementary Movie 2), the *blue* and *red arrows* point to the Cu_2_O/Cu interface step edge at 0 s and 0.5 s, respectively. Numbers 1, 2 and 3 mark the horizontal atomic planes at the Cu_2_O/Cu interface. *Scale bar*, 2 nm (**a**–**c**), 1 nm (**e**–**g**)

The Cu_2_O/Cu interfaces formed from the in situ oxidation are ideally suited for studying interfacial dynamics in a reducing environment. The Cu_2_O film is reduced at 350 °C by flowing H_2_ gas (*p*H_2_ = 4×10^−2^ Torr). Figures 2a–c show time-sequence HRTEM images of the Cu_2_O/Cu interface during the Cu_2_O reduction with the H_2_ flow (all the in situ TEM images are aligned to compensate for thermal drift). The red, green and blue dashed lines in Figs. 2a–c outline the location of the Cu_2_O/Cu interface. Detailed tracing of the movement of the Cu_2_O/Cu interface is depicted in Fig. 2d, where the relative positions of the interface at different times are shown for comparison. The Cu_2_O/Cu interface is initially observed to exhibit a wide and flat shape, with the presence of atomic steps at the two corner areas (Fig. 2a). As the reaction progresses, the Cu_2_O/Cu interface is observed to migrate toward the Cu_2_O side as the Cu_2_O is converted into metallic Cu along the Cu_2_O/Cu interface (Figs. 2b, c). The Cu_2_O/Cu interface maintains the wide and flat morphology and displays an overall normal movement toward the oxide. The interface is measured to migrate by the amount of ~8*d*_Cu(200)_ (~1.4 nm) toward the Cu_2_O layer during a time elapse of 27 s (Supplementary Movie 1). Meanwhile, the atomic steps at the Cu_2_O/Cu interface tend to smooth out quickly, thereby maintaining the sharply defined and microscopically flat interface during the course of the Cu_2_O → Cu conversion, i.e., the three atomic layer steps on the left corner of the interface (Fig. 2a) and a single atomic step in the middle of the interface (Fig. 2b), as indicated by the black arrows, are seen to disappear quickly.

Figures 2e–g show snapshots of the Cu_2_O → Cu interface transformation at a higher magnification view. It can be seen that the Cu_2_O → Cu conversion starts from the atomic steps at the corner region and propagates laterally via step-flow along the Cu_2_O/Cu interface. Figure 2e shows three atomic steps of Cu residing at the Cu_2_O/Cu interface, as indicated by the blue arrows. The blue arrows in Fig. 2f point to the same positions as shown in Fig. 2e, the red arrows point to the new locations of the steps after a time interval of 0.5 s. All the three steps are observed to sweep simultaneously and laterally along the in-plane direction (<100>) of the interface. In the subsequent 1.5 s (Fig. 2g), step 1 meets with the Cu step propagating from the right side to form a continuous Cu layer at the interface. Therefore, the Cu_2_O → Cu interface conversion occurs via the lateral flow of the atomic steps of the Cu substrate, with a coordinated retraction of the Cu_2_O at the step edges of the metal and oxide. This interfacial transformation is observed to start at the atomic steps of the Cu_2_O/Cu interface, proceed by lateral propagation, and merge with the metallic atomic steps initiated from the other corner area, thereby demonstrating that the step-edge controls the interface reaction. Meanwhile, the epitaxial Cu_2_O/Cu interface is maintained because the Cu substrate provides a structure template for the epitaxial growth of new Cu released from the reducing Cu_2_O.

### Grain rotation at the Cu_2_O/Cu interface

**Fig. 3:**
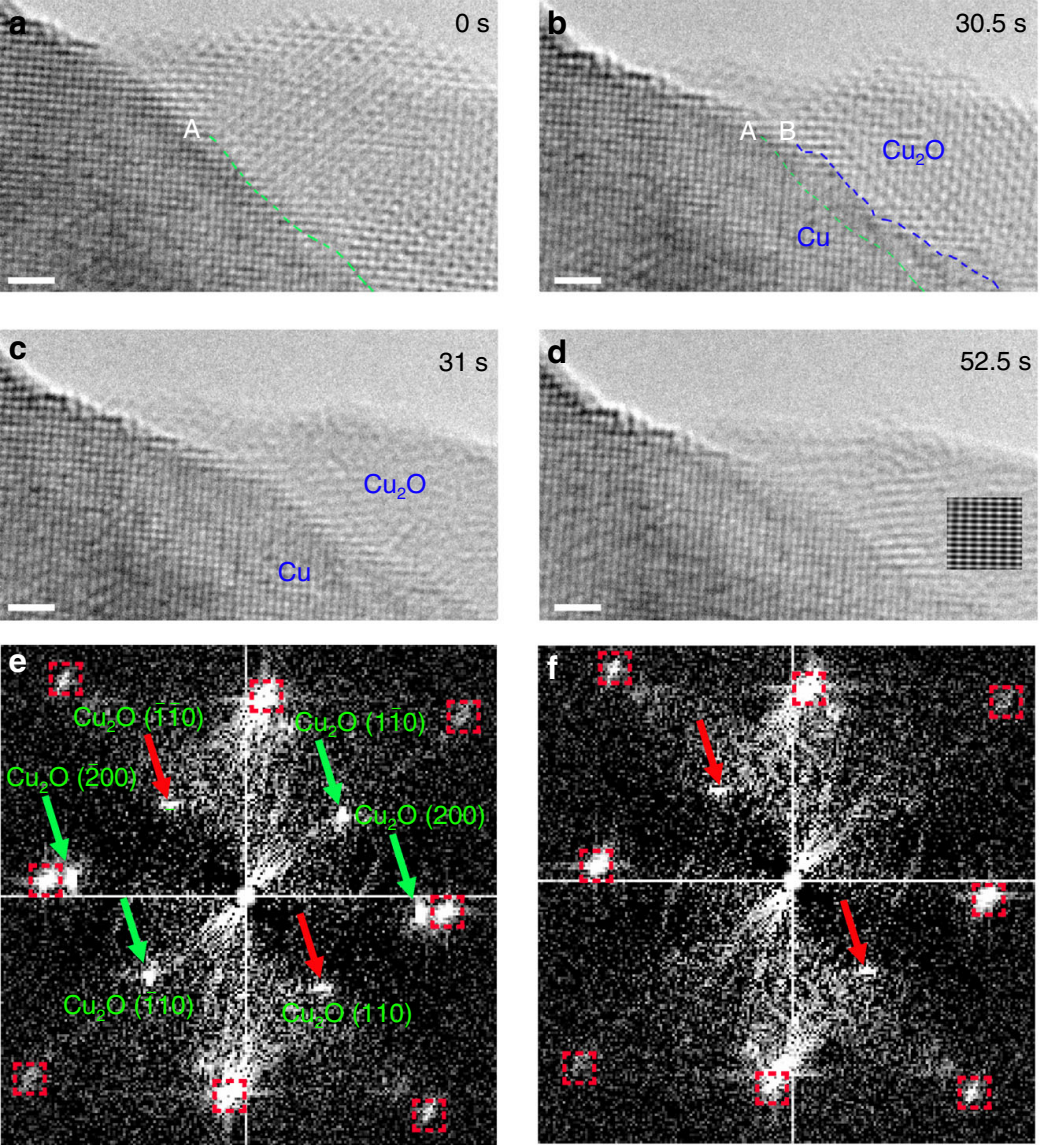
Cu_2_O island rotation along the Cu_2_O/Cu interface. **a**–**d** Time-sequence HRTEM images of the Cu_2_O→Cu reduction at *T* = 350 °C and *p*H_2_ = 4 × 10^−2^ Torr (Supplementary Movie 3). *Dashed lines* A and B mark the locations of the Cu_2_O/Cu interface at 0 s and 30.5 s, respectively. The *inset* in **d** is a simulated HRTEM of the Cu_2_O grain tilted by 33° with respect to the <001> direction. **e**, **f** Diffractograms of HRTEM images **b** and **c**, *green* and *red arrows* mark the Cu_2_O diffraction spots that disappear and remain, respectively, due to the rotation of the Cu_2_O island; the *red dashed squares* mark the Cu diffraction spots, which remain unchanged during the time interval. *Scale bar*, 1 nm

Continued reduction of the Cu_2_O results in a transition from an epitaxial to a non-epitaxial Cu_2_O/Cu interface via the rotation of Cu_2_O grains. Figure 3 shows an in situ TEM observation of how oxide reduction induces grain rotation with continued H_2_ exposure. Figure 3a marks the starting point (0 s) for this time sequence (the sample has been reduced a while before we move to this area for TEM imaging). The epitaxial relation of the Cu substrate and the Cu_2_O grain is Cu_2_O(200)//Cu(200) and Cu_2_O[001]//Cu[001], as identified from the [001]-zone axis HRTEM images shown in Figs. 3a, b. The Cu_2_O → Cu transformation that results in the propagation of the Cu_2_O/Cu interface into the Cu_2_O side while maintaining the epitaxial interface is observed between 0 and 30.5 s, where the interface propagation direction is accurately determined by measuring the change in the lattice spacing (Supplementary Note 3). The dashed lines in Fig. 3b mark the locations of the Cu_2_O/Cu interface at *t* = 0 s and *t* = 30.5 s, which clearly show that the Cu lattice grows toward the oxide by consuming the oxide via the interface-controlled process identified in Fig. 2. However, the lattice fringe contrast in the Cu_2_O grain shows a sudden change between Figs. 3b, c within just 0.5 s of the time interval. The clearly resolved two-dimensional lattice fringes of the Cu_2_O grain seen in Fig. 3b turn into the one-dimensional lattice fringes, as seen in Figs. 3c, d. In contrast, the lattice fringe contrast in the Cu region remains the same over the time, which rules out a possible effect from sample tilting with respect to the incident electron beam during the TEM imaging. Meanwhile, by comparing Figs. 3b, c, it can be seen that the projected area of the Cu_2_O grain becomes smaller within the short time interval. After grain rotation, the Cu_2_O grain loses its epitaxy with the Cu substrate: thus it no longer presents a zone-axis orientation with respect to the e-beam. Thus, the Cu_2_O/Cu interface transformation cannot be resolved clearly from the TEM imaging (Fig. 3c, d).

We first identify the cause for the sudden change of the lattice fringe contrast in the Cu_2_O grain between Figs. 3b, c. Given the apparent loss of the sharp lattice fringe contrast, two possibilities are considered. One possibility is related to a change in the crystal structure of the oxide island: however, this is ruled out by HRTEM image simulation of oxygen deficient Cu_2_O structures (Supplementary Note 4). The other possibility is that the entire Cu_2_O grain rotates, as induced by the Cu_2_O → Cu interfacial reduction in such a way that the Cu_2_O grain has deviated from the original <001> zone axis orientation. Figure 3e is a diffractogram of the HRTEM image of Fig. 3b, in which the [001]-zone-axis patterns of both Cu and Cu_2_O are clearly visible. Fig. 3f is the diffractogram of the HRTEM of Fig. 3c, in which the two {110} spots from the Cu_2_O island are still visible (indicate by the red arrows), confirming that the oxide island still remains the Cu_2_O structure, while its zone-axis orientation has tilted away from the original <001> direction. The new zone axis for the Cu_2_O grain is determined to be <−*a a* 1> by the Weiss zone law and the misorientation angle between the Cu substrate and the Cu_2_O grain can be quantitatively estimated as 33° from the relation of the projection areas of the Cu_2_O grain before and after the rotation (Supplementary Note 5). The simulated diffraction pattern (Supplementary Fig. 6) from the tilted Cu_2_O grain has a good match to the diffractogram shown in Fig. 3f, where only diffraction spots from {110} planes are present. We then perform HRTEM image simulation using the Cu_2_O model with an incident beam along the <−0.465 0.465 1> direction, which corresponds to the Cu_2_O rotation out of the (001) plane by 33° with respect to the <001> zone axis. Inset in Fig. 3d is a simulated HRTEM image along the <−0.465 0.465 1> zone axis of Cu_2_O, which shows the one-dimensional lattice fringe contrast, and again has good agreement with the experimental HRTEM image.

## Discussion

**Fig. 4:**
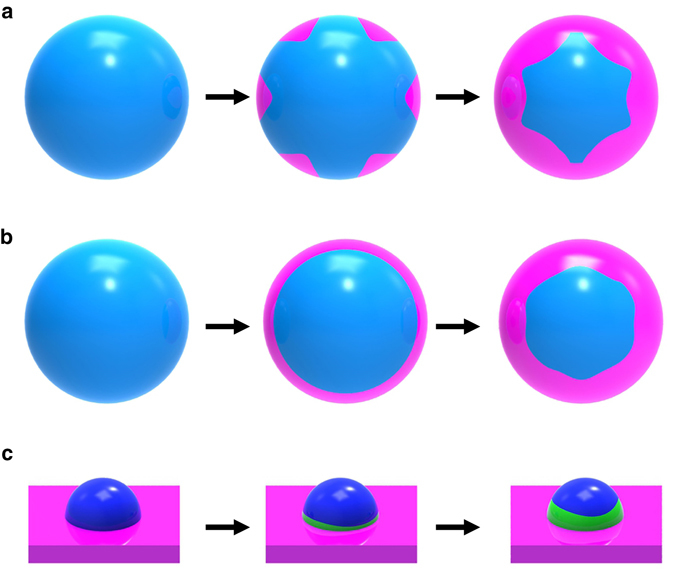
Schematic comparison of the oxide reduction models. **a** The nucleation and growth model, generation of small nuclei (denoted by *pink color*) of the reduced phase (e.g., a lower valence state oxide or pure metal) occurs on the parent oxide (*blue*) and the reaction interface increases until growing nuclei coalesce and then decreases. **b** The interface model, rapid formation of a uniform layer of a reduced phase (*pink*) on the parent oxide (*blue*) occurs very soon after contacting with a reducing gas. The reaction interface advances inward uniformly as the reaction proceeds, resulting in a spherical core of the oxide that shrinks with time. **c** Reduction of an oxide island (*dark blue*) on a metal support (*pink*), formation of the reduced phase (*green*) occurs preferentially at the buried metal/oxide interface

The reduction of metal oxides has traditionally been described using the nucleation and growth model or the interface model^22–24^. In the nucleation and growth model, the generation of small nuclei of the metallic phase occurs on the parent oxide, the oxide/metal interface advances inward and the reaction interface increases until the growing metal nuclei overlap, as shown schematically in Fig. 4a. In the interface model, the rapid formation of a uniform and continuous layer of the metallic phase on the parent oxide occurs and the reaction boundary moves inward as the reaction proceeds (Fig. 4b). While these phenomenological models have been found useful in the description of the reduction of bulk oxides^25, 26^, here we find that the reduction of supported Cu_2_O islands does not follow either the “nucleation and growth” or “interface” model. Rather than forming metallic Cu on the outer surface of the Cu_2_O islands, our in situ TEM observations demonstrate that the reduction of Cu_2_O to Cu occurs at the buried Cu_2_O/Cu interface (Fig. 4c), where Cu atoms in the parent oxide are directly dislodged to the step edges of the Cu lattice, resulting in step-flow-controlled dynamics. It can be seen that the Cu_2_O reduction at the existing Cu_2_O/Cu interface still involves the nucleation and growth of Cu at various sites of the Cu_2_O/Cu interface, which lead to a rough Cu_2_O/Cu interface with the hill-and valley feature (Supplementary Fig. 7). The lateral step-flow along the interface results in the smoothened Cu_2_O/Cu interface (as shown in Figs. 2e–g). The thickness of the oxide layer examined in our experiment is typically less 20 nm (i.e., the distance from the Cu_2_O/Cu interface to the outer surface of the oxide island). Within this thickness range, we can monitor the structure evolution of the whole Cu_2_O grain using in situ HRTEM imaging. However, if the oxide layer is too thick, oxide reduction may also take place at the outer surface of the oxide by following the aforementioned phenomenological “nucleation and growth” model or the “interface” model for the reduction of isolated oxides. The critical thickness of the oxide layer that leads to the transition from oxide reduction at the buried metal/oxide interface to the traditional phenomenological models depends on temperature. A higher reaction temperature corresponds to a faster diffusion rate for both H atoms and O vacancies: a thicker oxide layer would be expected in this case. However, thicker oxides are problematic for HRTEM imaging, and thus are beyond our ability to image.

**Fig. 5:**
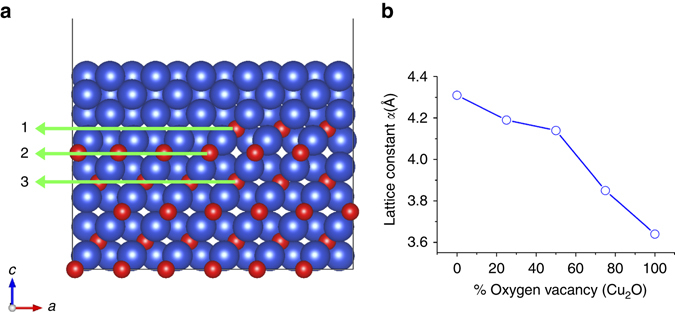
DFT calculations **a** Oxygen vacancy formation energy (*E*_vac_) for three representative interfacial sites, 1: interface step, 2: planar interface, 3: sub-interface. **b** Lattice constant as a function of the concentration of oxygen vacancies in Cu_2_O. *Blue* and *red spheres* represent Cu and O atoms, respectively

Microscopically, the reduction of metal oxides is a reaction caused by removing lattice oxygen, followed by a subsequent structural rearrangement of the parent lattice. In our case, the reducing species (H_2_ molecules) adsorb on the oxide surface and then dissociate into H atoms that may react directly with surface oxygen and/or further penetrate into the oxide and react with lattice oxygen to form hydroxyls. Because of the relatively high temperature (350 °C) employed in our reduction experiments, it is reasonable to expect that hydroxyls exist only as an intermediate and quickly desorb from the surface as H_2_O molecules, which leaves behind empty lattice sites (e.g., oxygen vacancies) in the oxide. Evaluation of the reduction activity can be based on the energy needed for removing lattice oxygen, which is defined as vacancy formation energy. Depending on the different sites such as the surface or interface, the oxygen vacancy formation energy can be different, which results in the oxide reduction along the minimum energy path. One would expect the oxide to be stable if oxygen vacancy formation is an energetically costly process. For example, a planar oxide surface usually remains relatively stable when exposed to a low H_2_ pressure^24^. In contrast, oxygen atoms may be easily removed at defective sites, i.e., surface steps. To check the stability of lattice O at the metal/oxide interface, we utilized density functional theory (DFT) to investigate energetics of Cu_2_O reduction by calculating oxygen vacancy formation energies at three representative sites near the Cu_2_O/Cu interface, i.e., an interface step edge, an interface terrace, and a sub-interface layer, as marked by 1, 2, and 3 in Fig. 5a. These are found to be 1.64, 2.1, and 1.98 eV, respectively (Supplementary Note 7). The interface step edge site shows a smaller oxygen vacancy formation energy compared to the planar interface or the sub-interface sites. These DFT results confirm that the oxide reduction along the interface step edge is preferred, consistent with the TEM observation of the step-flow growth of new Cu layers at the Cu_2_O/Cu interface during the initial stages of the oxide reduction, as shown in Fig. 2.

**Fig. 6:**
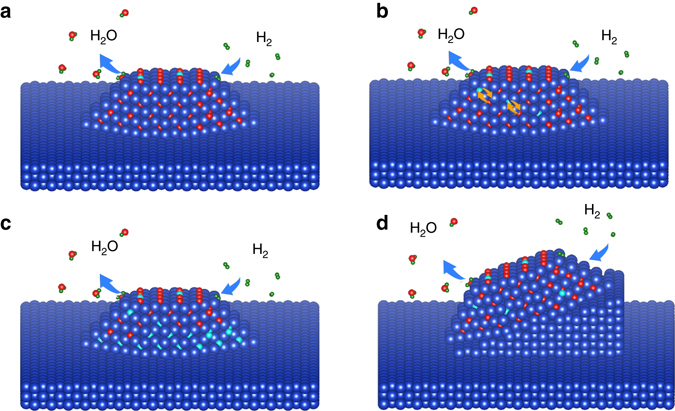
Pictorial illustration of the Cu_2_O island rotation. **a** Reaction of H_2_ molecules with lattice oxygen in Cu_2_O forms H_2_O molecules that desorb from the surface, leaving behind oxygen vacancies. **b** Oxygen vacancies migrate through the oxide layer toward the Cu_2_O/Cu interface. **c** Oxygen vacancies accumulate near the Cu_2_O/Cu interface region. **d** Abrupt collapse of the Cu_2_O lattice in the interface region into Cu results in the formation of a stepped Cu plateau, which drives the rotation of the entire Cu_2_O island. *Blue*, *red*, *green* and *cyan spheres* represent Cu, O and H atoms, and O vacancies, respectively

While Cu_2_O is reduced to Cu at the Cu_2_O/Cu interface, oxygen vacancies can be also generated at the oxide surface (Fig. 6a) because it is the first place for H_2_ adsorption and dissociation. The oxygen vacancies generated at the surfaces can migrate toward the Cu_2_O/Cu interface via exchange with adjacent oxygen atoms (Fig. 6b). When the Cu_2_O → Cu interfacial conversion does not keep up with the incoming flux of oxygen vacancies, oxygen vacancies then accumulate at the Cu_2_O/Cu interface (Fig. 6c). This is also apparent in our in situ TEM observations, which show that no structural changes are detected in the bulk of the oxide while the interface is undergoing the Cu_2_O → Cu conversion (Figs. 2 and 3). This indicates that the bulk has a much lower oxygen vacancy concentration when compared to the Cu_2_O/Cu interface region. This is consistent with our DFT calculation (Fig. 5b), which shows that the lattice parameter of the oxide has a larger tendency to collapse into the Cu lattice when the oxygen vacancy concentration reaches a critical value (e.g., 50%). The abrupt collapse of the oxide lattice will result in the Cu_2_O/Cu interface decohesion. The rotation of the Cu_2_O grain shown in Figs. 3b, c) is induced by the anisotropic shrinkage of the Cu_2_O lattice, as illustrated in Fig. 6d. Cu atoms liberated from the collapse of the Cu_2_O lattice near the Cu_2_O/Cu interface region aggregate near the corner area, which results in the formation of a stepped Cu plateau at that location. As a result, the initially flat and epitaxial Cu_2_O/Cu interface becomes highly tilted, which provides the driving force for the grain rotation that is seen experimentally.

In summary, we use an in situ approach to create Cu_2_O/Cu interfaces by oxidation of Cu and subsequently monitor the metal/oxide interfacial transformation induced by the reaction with H_2_. Our real time, atomic-scale imaging of the dynamics of how Cu_2_O islands transform when subjected to a reducing gas environment demonstrates that the Cu_2_O reduction occurs initially via the layer-by-layer retraction of the Cu_2_O lattice, with the coordinated growth of the Cu lattice controlled by step-flow along the Cu_2_O/Cu interface. Continued accumulation of oxygen vacancies at the Cu_2_O/Cu interface region results in the abrupt collapse of the Cu_2_O lattice with the concomitant formation of a tilted Cu_2_O/Cu interface, which then drives the rotation of the Cu_2_O islands. These results demonstrate that the reduction dynamics of Cu_2_O islands are governed by atomic processes occurring at the Cu_2_O/Cu interface: an observation which is fundamentally different from the mechanisms known to be associated with the reduction of bulk or isolated oxides. Because the formation of oxide islands is a common phenomenon during the oxidation of metals, and because supported oxides are widely used in heterogeneous catalysis, our results reveal the unique role of the oxide/metal interfaces in controlling the reaction mechanism. These observations have broader implications for understanding and manipulating the way that buried interfaces can affect gas-surface reaction kinetics.

## Methods

### Sample preparation and in situ TEM characterization

Single-crystal Cu(100) thin films with a normal thickness of 50 nm were grown on NaCl(100) in a ultrahigh vacuum e-beam evaporation system. The as-prepared Cu thin film was then removed from the substrate by dissolving the NaCl in deionized water and mounted on a Si_3_N_4_ membrane window with 200-nm pores for TEM observation. In situ TEM experiments were performed using a dedicated environmental TEM (FEI Titan 80–300) equipped with an objective-lens aberration corrector. The microscope has a spatial resolution of 0.8 Å in the high-resolution TEM mode, even at the elevated temperature and pressure local to the sample.

### In situ TEM imaging of Cu_2_O/Cu interfacial transformation

The experiment involves three essential steps for the in situ formation of Cu_2_O/Cu interfaces and subsequent observation of the Cu_2_O/Cu interfacial transformation. The first step is annealing of a Cu thin film inside the microscope at 600 °C with H_2_ gas flowing at a pressure of 10^−3^ Torr for 20 min. This gradually both cleans and thins the sample, resulting in the formation of holes with faceted edges (dominated by (100) and (110) facets). These freshly created facets are atomically clean and ideal for subsequent oxide growth by exposing the sample to oxygen gas. The second step is therefore the creation of the Cu_2_O/Cu interface by oxidizing the faceted Cu edges. This is done by pumping the H_2_ out of the microscope, and then immediately providing oxygen gas at the pressure of *p*O_2_ = 5×10^−3^ Torr and *T* = 350 °C for 10 min. In order to observe the subsequent reduction of the newly formed oxide, the oxygen gas is pumped away, and then H_2_ gas is again inserted into the chamber. In situ TEM observation of the oxide reduction process is by imaging along the Cu_2_O/Cu interface under the flow of H_2_ gas at *p*H_2_ = 4×10^−2^ Torr and 350 °C. To overcome any potential e-beam effects (i.e., to rule them out as factors affecting the in situ TEM results and to ensure that we have studied the intrinsic behavior of the oxide reduction), we employed a first-of-its kind direct electron camera (K2) which allows for fast image acquisition (400 frames per second) with a significantly reduced electron dose rate. We examined the possible effect of electron beam irradiation by comparing the oxide reduction in areas that had been irradiated with electron beam and different sample areas that had not. The in situ TEM images given in Supplementary Note 8 exemplify these “comparison” experiments, which showed that the Cu_2_O islands with a thickness of ~10 nm from the Cu_2_O/Cu interface to the outer surface can be reduced completely to metallic Cu after 5 min of H_2_ gas flow at *p*H_2_ = 1×10^−3^ Torr and *T* = 350 °C, irrespective of whether the e-beam was irradiating the sample or not. Conducting experiments of this type to understand the effect of electron irradiation is part of our experimental protocol, and in this case ensured that electron irradiation has a negligible effect on the observed oxide reduction process.

### DFT calculations

DFT calculations are performed using the Vienna ab-initio simulation package^27–29^ with the PW91 generalized gradient approximation^30^ and projector augmented wave^31^ potentials. Our previous works have confirmed that a cutoff energy of 380 eV is sufficient to give a well converged system energy^32^. The Brillouin-zone integration is performed using (4 × 4 × 1) *K*-point meshes based on Monkhorst-Pack grids^33^ and with broadening of the Fermi surface according to Methfessel-Paxton smearing technique^34^ with a smearing parameter of 0.2 eV. The interface model is simulated by placing the interface between four layers of Cu(100) and four layers of Cu_2_O(100) as shown in Fig. 5a. Each Cu layer in Cu and Cu_2_O contains eight Cu atoms, and each O layer in Cu_2_O contains four O atoms, while the step interface contains two O atoms. The top layer of Cu and the bottom layers of Cu_2_O are fixed at the lattice position and all other atoms are allowed to fully relax during optimization until all force components acting on the atoms are below 0.02 eV/Å. Successive slabs are separated by a vacuum region of at least 13 Å.

The oxygen vacancy formation energy *E*_vac_ is calculated using the following equation

1 $${E_{{\rm{vac}}}} = {E_{{\rm{tot}}}} - {E_{{\rm{ref}}}} + \frac{{{N_0}}}{2}{E_{{{\rm{O}}_2}}},$$

Where, *E*_tot_ is the total energy of the Cu-O system containing *N*_o_ number of oxygen vacancies, *E*_ref_ is the energy of the structure without the oxygen vacancies, and ${E_{{{\rm{O}}_2}}}$ is the energy of an isolated oxygen molecule. The atomic structures are visualized using the VESTA package.

There are two different approaches used to study the interface formed by the two phases. The first one, called the (1 × 1) model, uses one unit cell in the plane of the interface for each phase and is used for phases with a relatively small mismatch. The lattice parameters are scaled until the phases of the interface match perfectly. This is the primary method used in first principles studies of interface models^35–38^. The second approach consists of determining the ratio of two integers that is closest to the ratio of lattice parameters in the interface. This method leads to incoherent or semi-coherent interface models with minimized mismatch^35^.

In the case of Cu and Cu_2_O phases, the ratio between the lattice parameters of Cu to Cu_2_O is ~0.84, Cu and Cu_2_O having calculated bulk lattice parameters of 3.64 and 4.31 Å, respectively. Using the second approach, this ratio could be approximated by 5/6, with an interface model (5 × 5)Cu_2_O /(6 × 6)Cu. Previous studies have shown that the epitaxial growth of Cu_2_O thin films on Cu substrates results in a (5 × 6) coincidence site lattice at the Cu_2_O/Cu interface^6^. This interface model significantly reduced the strain to ~1.48%. However, in performing DFT calculations of the oxide islanding during oxidation, the system size reaches up to 516 total atoms. Such an interface model is too large and computationally expensive for many DFT calculations. Wang et al.^39^compared both approaches in a study of Si/Cu interfaces perpendicular to the [111] direction. Even with a large mismatch of 35% between Si and Cu, they showed that a full relaxation of a (1 × 1) interface model only overestimates the work of separation of the interface (2 × 2)Si/(3 × 3)Cu model by ~3%. Regarding the Cu_2_O/Cu interface, a previous study also adopted the (1 × 1) model for calculations on the Cu_2_O/Cu interfaces perpendicular to the [111] direction. Therefore, the (1 × 1) model can be used as a reasonable approach for the Cu_2_O/Cu interface perpendicular to the [100] direction.

### Data availability

All data generated or analyzed during this study are included in this published article (and its supplementary information files).

## Electronic supplementary material

Supplementary Information

Supplementary Movie 1

Supplemental Movie 2

Supplemental Movie 3
