# Supplementary material for: In situ atomic-scale imaging of the metal/oxide interfacial transformation
Source: Nat Commun. 2017 Aug 21;8:307. doi: 10.1038/s41467-017-00371-4 (PMC5563505; doi:10.1038/s41467-017-00371-4)
Supplement: Supplementary file 1 — Supplementary Information [file 41467_2017_371_MOESM1_ESM.pdf]

SI GUIDE

File Name: Supplementary Information
Description: Supplementary Figures, Supplementary Notes and Supplementary References.

File Name: Supplementary Movie 1
Description: (the movie from which Figs. 2(a-c) are extracted): *In situ* TEM movie showing the Cu_2O→Cu conversion at the Cu_2O/Cu interface at T = 350 ºC and pH_2 = 4×10^{-2} Torr.

File Name: Supplementary Movie 2
Description: (the movie from which Figs. 2(e-g) are extracted): *In situ* TEM movie showing the atomic process of the Cu_2O→Cu interfacial conversion at T = 350 ºC and pH_2 = 4×10^{-2} Torr.

File Name: Supplementary Movie 3
Description: (the movies from which Fig. 3 is extracted): *In situ* TEM movie showing the Cu_2O reduction induced Cu_2O grain rotation at T = 350 ºC and pH_2 = 4×10^{-2} Torr

Supplementary Note 1. Identification of Cu and Cu_2O

The atomic spacing of a half unit cell in the substrate region is measured to be 1.8 Å (Fig. 1b), which is in agreement with the d_{200} spacing of Cu lattice. From FFT analysis of the overall area, we notice that there are two sets of diffraction patterns of 45° with respect to each other as shown by the inset in Fig. 1b. One set of the diffraction patterns is highlighted with the green lines and matches with Cu_2O. The other set of diffraction spots are marked by purple rings and match well with Cu. By comparing the relative angle and position of diffraction spots of the FFT pattern with the simulated diffraction pattern of Cu and Cu_2O shown below (Supplementary Fig. 1), we can identify unambiguously these two patterns as Cu and Cu_2O phases, thereby confirming the oxide phase as Cu_2O.

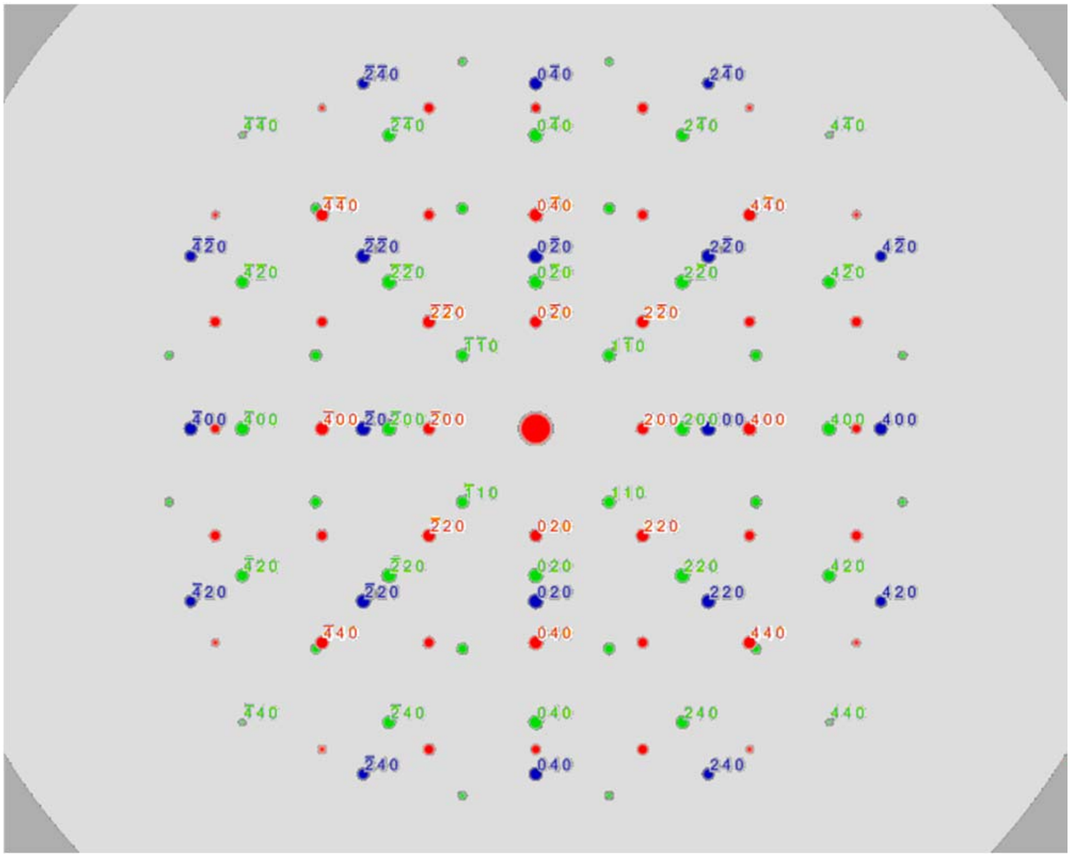

Supplementary Figure 1: **Simulated [001] zone axis electron diffraction patterns of the Cu and its oxides (Cu_2O, Cu_4O_3) and their indexing.** Blue, green and red dots represent the superposed diffraction spots from Cu, Cu_2O and Cu_4O_3, respectively.

Supplementary Note 2. Atomistic models of the Cu_2O/Cu interface

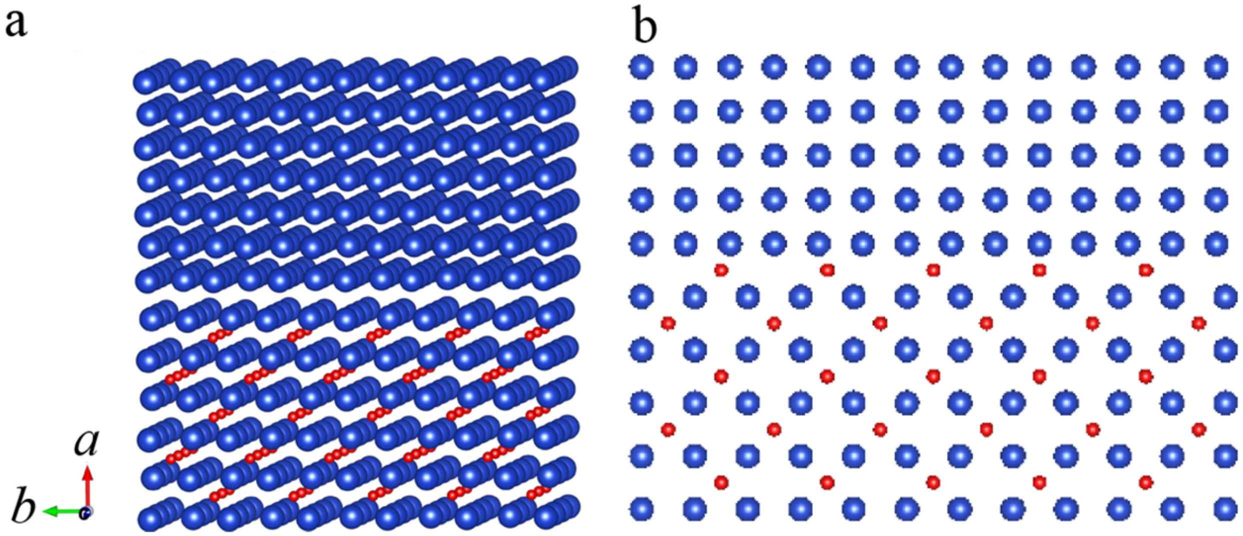

Supplementary Figure 2: Structure model of the interface matching between Cu and Cu_2O. Blue and red spheres represent Cu and O atoms, respectively. (a) 3D view of the Cu_2O/Cu interface, (b) [001] projected view of the Cu_2O/Cu interface.

Supplementary Note 3. Identification of the propagation direction of the Cu_2O/Cu interface

The moving direction of the Cu front is determined by quantitatively measuring changes in the lattice spacing across the Cu_2O/Cu interface. As an example, we use the two HRTEM image frames from Supplementary Movie 3. Supplementary Fig. 3 is a zoomed-in view of the Cu_2O/Cu interface region. We can randomly pick up a lattice point (marked with a white dot in Supplementary Fig. 3(a)) in the Cu area as the marker and then track this lattice point frame by frame (from Supplementary Movie 3). In this way, the direction of the Cu_2O/Cu interface propagation can be determined. As each of the lattice position is relatively clear at the boundary area, we use the lattice spacing to accurately locate the Cu_2O/Cu interface. We mark each of the lattice points with orange rings (Supplementary Figs. 3(a, b)) above the marker along the vertical direction towards the Cu_2O region. We then measure each of the d_{200} lattice spacing along the vertical direction, and the results are given in Supplementary Table 1. d_{mn} (m = 1, 2, 3, 4, 5, 6, n = 1, 2, 3, 4, 5, 6) in Supplementary Table 1 stands for the lattice spacing between the mth and n^{th} horizontal atomic planes numbered in Supplementary Fig. 3. As we can see from Supplementary Table 1, the abrupt jump of the lattice spacing from $d_{\mathrm{Cu_2O(200)}}$ (2.13 Å) to $d_{\mathrm{Cu\ (200)}}$ (1.8 Å) allows for determining the location of the Cu_2O/Cu interface. Using this approach, we can identify that the Cu_2O/Cu interface is between atomic planes 5 and 6 for the frame at 0 s (as pointed by the red arrow in Supplementary Fig. 3(a)), which is one $d_{\mathrm{Cu(200)}}$ spacing from the marker (lattice point 6 in Supplementary Fig. 3(a)). In Supplementary Fig. 3(b), the Cu_2O/Cu interface can be identified at between atomic plane 3 and 4, which is three $d_{\mathrm{Cu\ (200)}}$ (1.8 Å) from the same marker (lattice point 6 in Supplementary Fig. 3(b)). Therefore, the lattice spacing analysis indicates that the Cu_2O/Cu interface actually propagates towards the Cu_2O region by two $d_{\mathrm{Cu\ (200)}}$ spacings during the time interval of the two image frames.

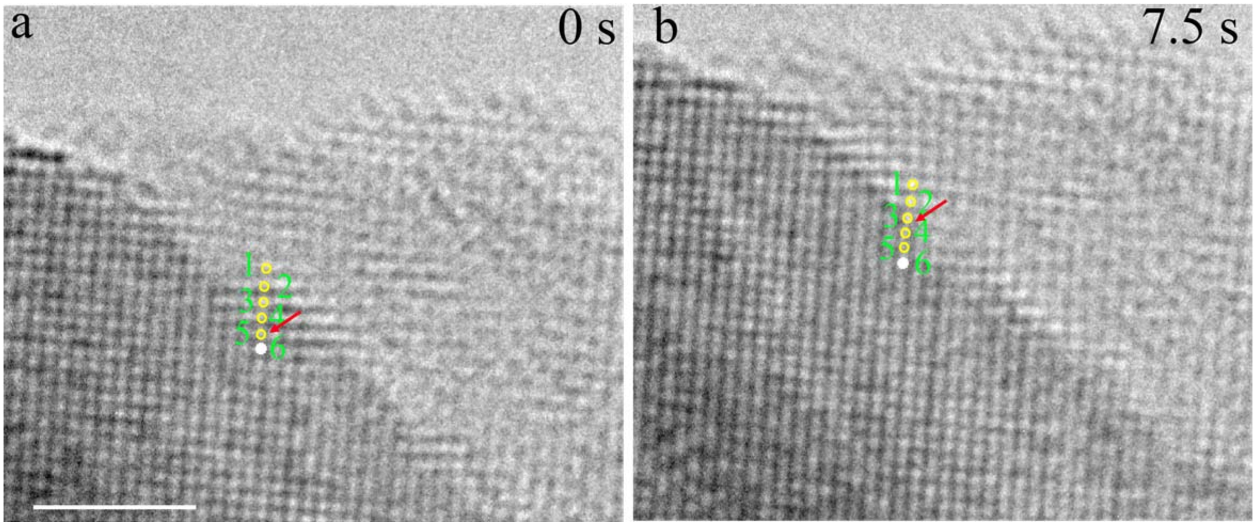

Supplementary Figure 3: Analysis of the Cu_2O/Cu interface propagation direction. The white dots (numbered as 6) in (**a**) and (**b**) correspond to the same lattice point in the two image frame by tracking this lattice point frame by frame in Supplementary Movie 3, which serves as a marker to identify the moving direction of the Cu_2O/Cu interface. Numbers 1-5 label the lattice points above the marker along the vertical direction toward the Cu_2O region. The d_{200} lattice spacings along the marked atomic planes are measured and listed in Supplementary Table 1. The red arrows in (**a**) and (**b**) point to the migration of the location of the Cu_2O/Cu interface along the marked vertical direction, which correspond to atom plane 5 in (**a**) and atom plane 3 in (**b**), respectively. Scale bar, 2 nm.

Supplementary Table 1. Lattice spacing between each lattice point labeled by the orange rings in Supplementary Fig. 3.

	d_{12} (Å)	d_{23} (Å)	d_{34} (Å)	d_{45} (Å)	d_{56} (Å)
Supplementary Fig. 3(a)	2.10	2.08	2.09	2.10	1.81
Supplementary Fig. 3(b)	2.09	2.12	1.81	1.80	1.80

Supplementary Note 4. HRTEM simulation of Cu_2O lattice with different concentrations of oxygen vacancies

As the Cu_2O grain is under the exposure of H_2, one of possibilities that might lead to the contrast change from Fig. 3(b) to Fig. 3(c) is the oxide reduction in the bulk. Removing oxygen atoms from the perfect lattice of Cu_2O will generate vacancies. The subsequent atomic relaxation around the vacancies results in the shifting of neighboring atoms, which may lead to the loss of the sharp lattice contrast. We tentatively deem the blur image contrast of the oxide phase (the Cu_2O lattice in Fig. 3(c)) as oxygen-deficient Cu_2O. We employ DFT to explore the optimized cell structures containing different concentrations of oxygen vacancies, and check if the relaxed structures could generate the strip contrast as shown in Fig. 3c in the HRTEM simulations. 25%, 50% and 75% randomly chosen oxygen atoms are taken out of the perfect Cu_2O cell to represent the presence of different concentrations of oxygen vacancies. DFT is then used to perform the structure optimization and obtain the fully relaxed cells with the different concentrations of oxygen vacancies. To create a larger structure for HRTEM image simulations, the DFT-relaxed cells are repeated in three dimensions. Supplementary Figs. 4(f-h) are the simulated HRTEM images based on the DFT-relaxed structure models shown in Supplementary Figs. 4(b-d), in which the green square outlines the unit cell obtained from the DFT calculations. The modulated contrast feature in the simulated HRTEM images is caused by the periodically repeating supercells. Simulated HRTEM images using the DFT-relaxed structures of the oxygen-deficient Cu_2O show sharp two-dimensional lattice fringe contrast (Fig. 3(d)), confirming that the one-dimensional fringe contrast is not induced by the oxide reduction in the bulk.

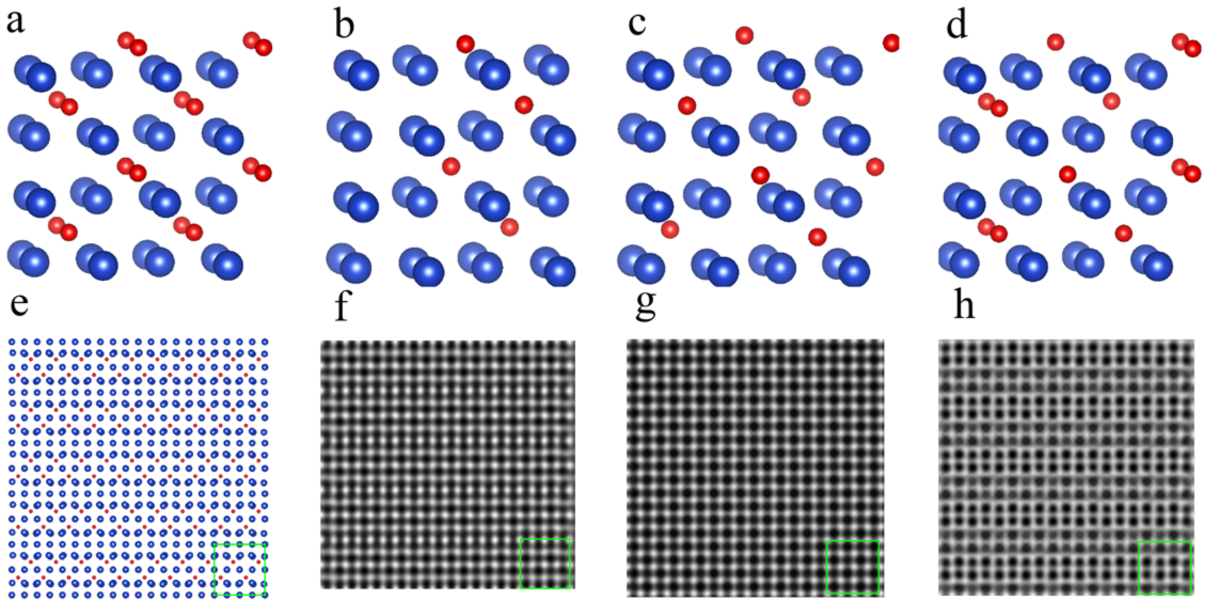

Supplementary Figure 4: Atomic structure and HRTEM simulated contrast of the oxygen deficient models. (a) Prefect Cu_2O structure. (b-d) The Cu_2O models containing oxygen vacancies of 75%, 50%, and 25% for **b, c** and **d**, respectively. (e)The expanded cell based on **b**. (f-h) HRTEM simulated contrast based on the expanded models of **b, c** and **d**, respectively. The green squares in (e-g) outline the unit cells from DFT calculations.

Supplementary Note 5. Electron diffraction simulation for Cu_2O grains deviated from <001>.

As shown in Fig. 3(f), the {110} diffraction spots are still visible after the island rotation, which allows for determining the new zone axis for the tilted Cu_2O grain. For a plane (*hkl*) with the zone [*UVW*], the Weiss zone law gives the equation: $hU + kV + lW = 0$. By substituting the *hkl* value for the (110) plane into the equation, h=1, k=1 and l=0, we have U+V=0. Therefore, the expression for the new zone axis is <-*a a* 1>.

Meanwhile, we can estimate the rotation angle by measuring the shrinkage of the projected area of the Cu_2O grain after its rotation. The areas outlined by the dashed white lines in Supplementary Figs. 5(a, b) are of 132393 and 110813 pixels, respectively, which give a ratio of ~ 0.84 of the projection areas before and after the grain rotation. The relationship between the projected areas before (A) and after (B) rotation is B=$A\cos\theta$, where θ is the rotation angle. By substituting B/A = 0.84 into this relation, we can find θ = 33°, which corresponds to the <-0.465 0.465 1> direction by the Cu_2O rotation out of the (001) plane by 33° with respect to the <001> zone axis.

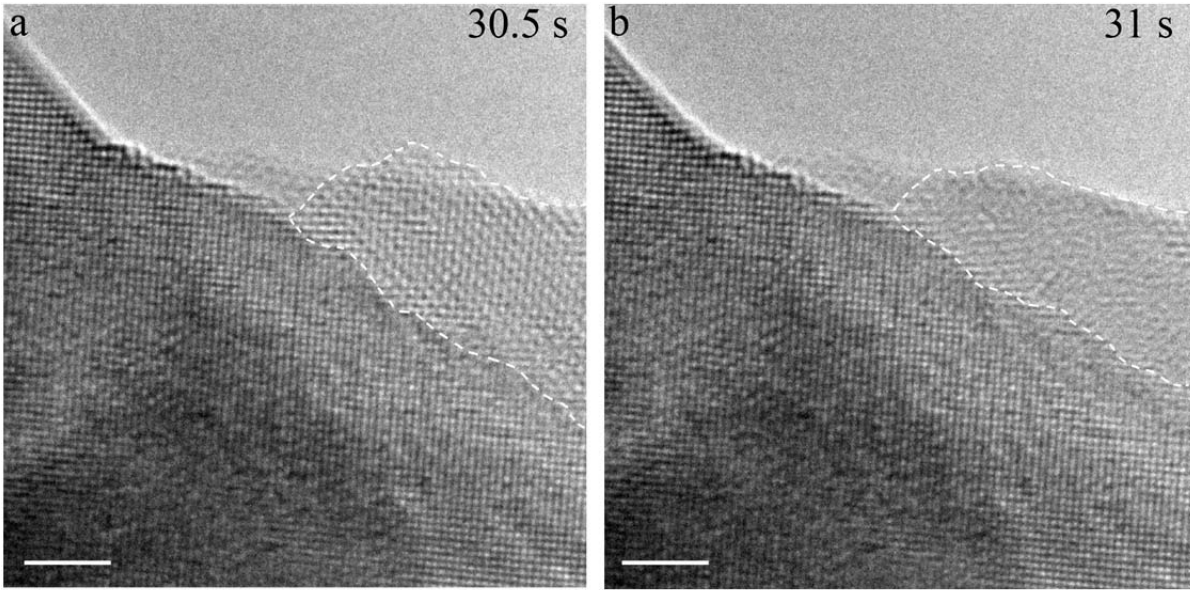

Supplementary Figure 5: Volume shrinkage during the Cu_2O grain rotation. The white dashed lines in (a) and (b) outline the projected area of the Cu_2O region before and after the island rotation. Scale bar, 2 nm.

We then simulate the electron diffraction pattern with the zone axis <-0.465 0.465 1>. Supplementary Fig. 6 is a simulated diffraction pattern of $\theta = 33^o$, which shows the presence of the (110)-type reflections near the (000) spot, consistent with the diffractogram (Fig. 3(f)) of the experimental HRTEM image obtained after the Cu_2O island rotation.

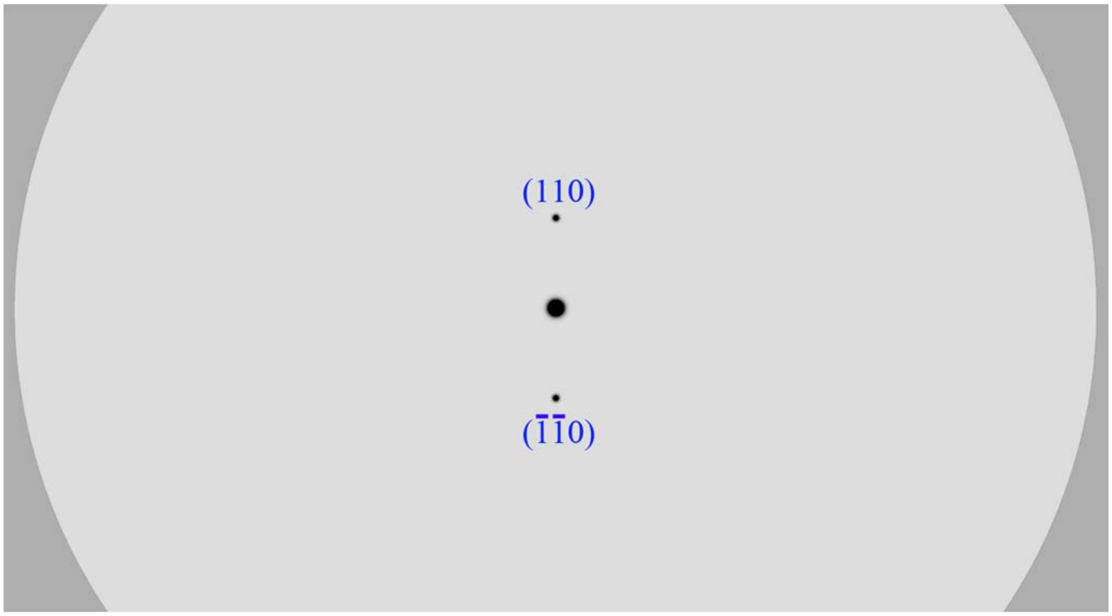

Supplementary Figure 6: **Electron diffraction simulation.** Simulated diffraction pattern of Cu_2O from the <-0.47 0.47 1> zone axes, which is 33.6^o deviated from the original <001> zone axis as determined from the island-rotation induced shrinkage in the projected area of the Cu_2O island.

Supplementary Note 6. Hill-and-valley feature at the Cu_2O/Cu interface during the Cu_2O reduction.

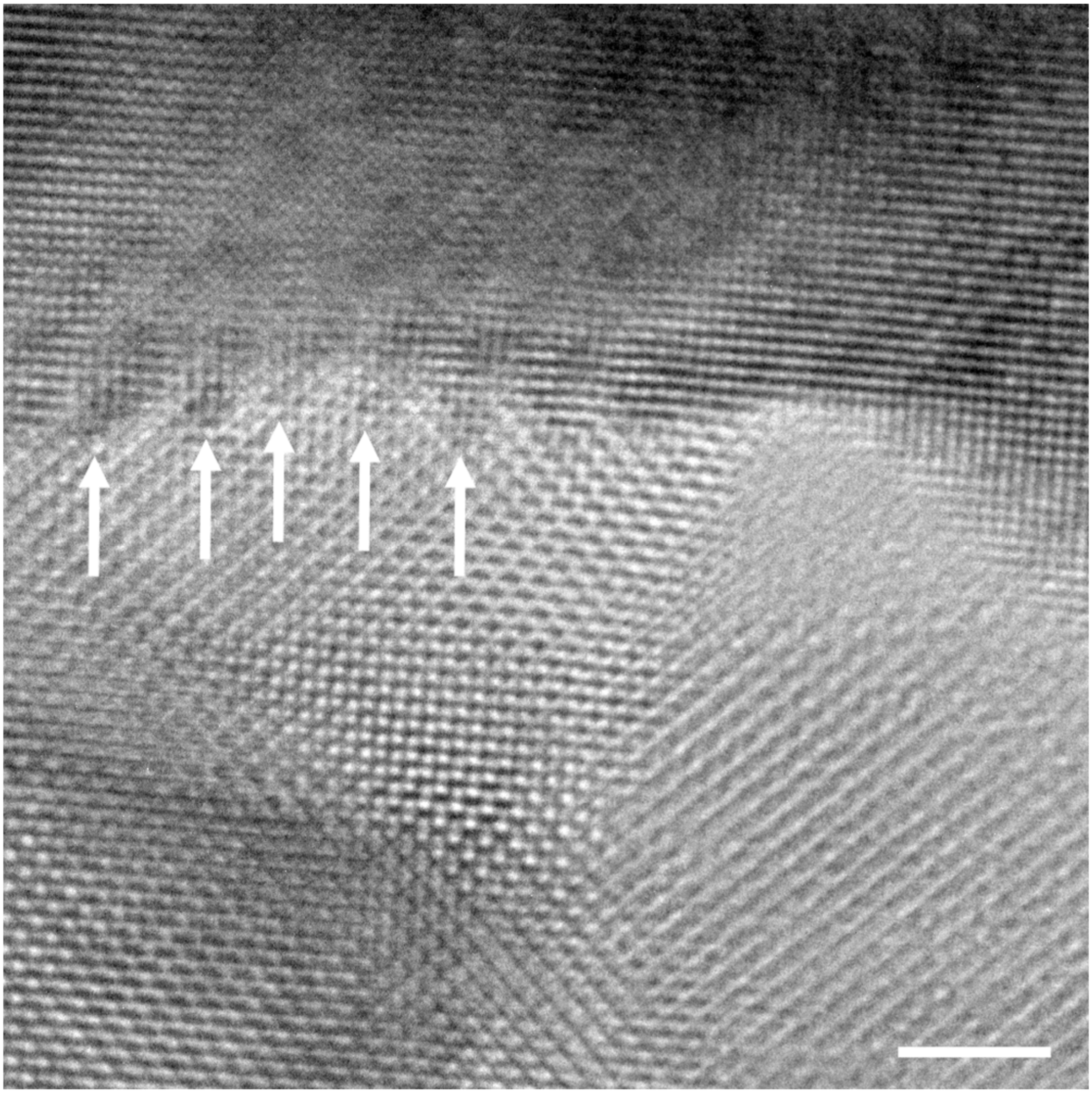

Supplementary Figure 7 | Cu_2O reduction at the Cu_2O/Cu interface. Formation of the hill-and valley feature at the Cu_2O/Cu interface by nucleation and growth of Cu at various sites of the Cu_2O/Cu interface during the reduction of a Cu_2O island on the Cu surface. Scale bar, 2 nm

Supplementary Note 7. DFT calculations of oxygen vacancy formation

Oxygen vacancy formation at the Cu_2O/Cu interface

In our (1×1) Cu_2O/Cu (100) interface model, shown in Supplementary Fig. 8(a), we expand the model to more than just one unit cell per phase in order to model our stepped-interface, keeping the 1:1 phase ratio. We determine the optimized lattice parameters for our interface model by fixing the b parameter while varying the c parameter (see Supplementary Fig. 8(a) for a, b, c directions), until the minimum energy is reached. Supplementary Fig. 8(b) shows the energy for each relaxation of different lattice parameters. For each *a*, *b* lattice parameter, there is a different *c* parameter. Since the atoms between the top and bottom of the slab are free to relax all directions, and there are two phases present, c cannot exactly define the lattice parameter for both phases. Rather, it is the scaling lattice parameter for the entire cell in the direction perpendicular to [100] and would be the average lattice parameter c throughout the cell. We start from a range of values around the bulk equilibrium lattice parameter values and report the energies close to the minimum. We determine that the optimized lattice parameters of our interface model are 3.93 Å for *a*, *b* and 4.31 Å for *c*. This causes the Cu region to be expanded in the *a*, *b* parameters, while the parameter of *a* in Cu_2O is compressed. The *c* parameter in the Cu phase is then compressed, while it is expanded for the Cu_2O phase.

The O vacancy locations are shown in Fig. 5(a), labeled as 1, 2 and 3. Before the removal of the O atom at the stepped interface, Site 1 has the lowest formation energy and smallest atomic average inward Cu atomic displacement. The Cu atoms surrounding Site 1 have the longest average Cu-O bond length of 1.91 Å. This is because of the increased planar spacing in Cu_2O. This site is the furthest from the Cu_2O/Cu interface and has the closest properties to bulk Cu_2O compared to the other 2 sites, having an average Cu-O bond length of 1.86 Å. Site 2 is located between the "bulk" Cu_2O and the stepped interface and has an average Cu-O bond length of 1.90 Å. Supplementary Table 2 shows the O vacancy formation energy as well as average inward Cu atomic displacement for all three sites. The inward Cu atomic displacement is an average of the 4 surrounding Cu atoms that make up the tetrahedral with the O atom located in its center. When the O atom is removed, the Cu atoms are displaced and the values for the

atomic displacement are determined by calculating the Cu-O distances before the O vacancy and then the Cu-O_{vac} distances after the O is removed, where O_{vac} is assumed to be located in the same position as the O atom. As the O vacancy is located further from the stepped interface, the inward Cu-atomic displacement decreases. The O vacancy formation energy is lowest at Site 1, thus making O atoms at the stepped interface most likely to be removed during the oxide reduction.

Oxygen vacancy in the bulk

Kim *et al.* [1] used DFT to study O vacancies in CuO leading to Cu, which requires an expansion of the metal lattice and a substantial change of the cell geometry. However, the metal sublattice in Cu_2O is similar to that of metallic Cu, so the formation from Cu_2O to Cu only requires a contraction of the lattice as O atoms are removed. Therefore, in our bulk calculations, we keep the *a*, *b* and *c* cell parameters equivalent to maintain the cubic structure while contracting the cell.

After removing 25%, 50% and 75% of the O atoms in bulk Cu_2O in an ordered fashion, so that there is at least one missing O atom in any O layer perpendicular to the <100> directions, we calculate the optimized lattice constant while maintaining the cubic shape. Fig. 5(b) shows the optimized lattice constant *a* as a function of % O vacancies. When 25% of the O atoms are removed from Cu_2O, the optimized lattice parameter only decreases by 3.00% and 50% O removal is only a 3.94% decrease. The lattice parameter is changed more drastically when 75% of the O atoms are removed and the composition is closer to pure Cu, resulting in a lattice parameter decrease of 10.67%.

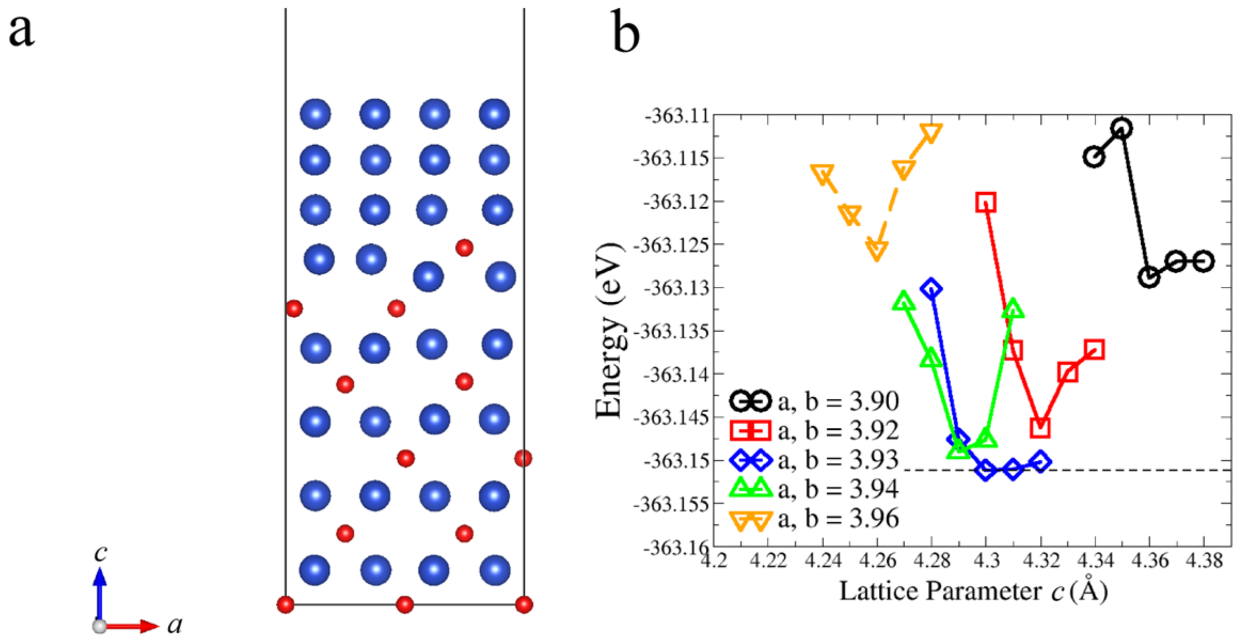

Supplementary Figure 8: Energy Profile for the interface model as a function of the *c* parameter. *a* and *b* are kept constant while relaxing the *c* parameter.

Supplementary Table 2: Oxygen vacancy formation energy and the average inward Cu atomic displacement for the Cu_2O/Cu interfacial sites shown in Fig. 5(a)

Site	Oxygen Vacancy Formation Energy (eV)	Average Inward Cu Atomic displacement (Å) / (%)
1	1.64	0.32 (16.87)
2	2.10	0.33 (17.52)
3	1.98	0.34 (18.62)

Supplementary Note 8. Excluding e-beam effect on the metal-oxide interface reduction

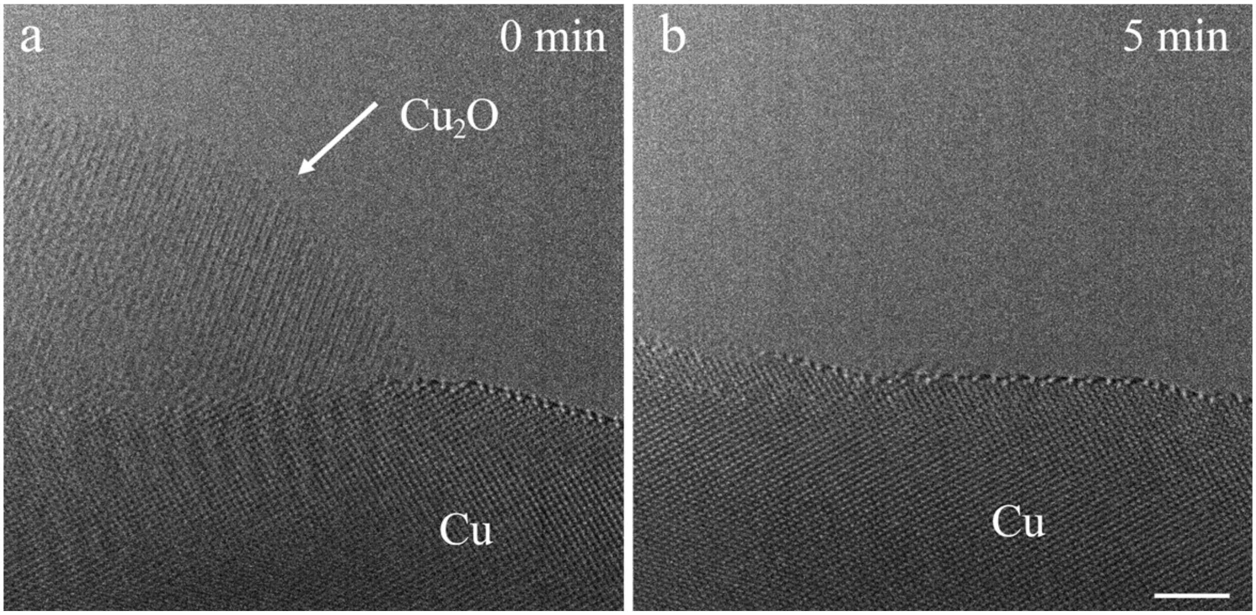

Supplementary Figure 9: Cu_2O reduction with low electron beam dose. The reduction of a Cu_2O island on a Cu surface with the flow of H_2 gas at $pH_2 = 1\times10^{-3}$ Torr and T = 350 °C. To avoid possible long electron beam exposure induced oxide reduction, the beam was on only when during the very brief, low-dose exposures used to take the TEM images with a direct electron detector. Scale bar, 2 nm.

Supplementary References

[1] J. Y. Kim, J. A. Rodriguez, J. C. Hanson, A. I. Frenkel and P. L. Lee, *J. Am. Chem. Soc.* **125**, 10684-10692 (2003).
